# Characterization of the mitochondrial genome of *Chlorolobion braunii* ITBB-AG6, an azolla-associated green alga isolated from sanitary sewage

**DOI:** 10.1080/23802359.2023.2241573

**Published:** 2023-08-03

**Authors:** Yiliang He, Shuai Ma, Qiaoqiao Yang, Huanggui Lai, Jiaming Zhang

**Affiliations:** a College of Agriculture, Hainan University, Haikou, China; b Institute of Tropical Bioscience and Biotechnology, Hainan Key Laboratory of Microbiological Resources, Hainan Bioenergy Center, Chinese Academy of Tropical Agricultural Sciences, Haikou, China

**Keywords:** *Chlorolobion braunii*, mitochondrial genome, phylogenetic analysis, organelle genome

## Abstract

Sphaeropleales have the characteristics of rapid growth, high oil content, and efficient removal rates of nitrogen and phosphorus in sewage waters, and is potentially valuable in biodiesel production and environmental remediation. In this study, we isolated a strain of Sphaeropleales, *Chlorolobion braunii* strain ITBB-AG6 from an azolla community in a sewage pond. Its mitochondrial genome contains 110,124 bp and harbors at least 40 genes, including 15 protein-coding genes, 20 tRNA genes, and three rRNA genes. The protein-coding genes include two for ATP synthases, seven for NAD(P)H-quinone oxidoreductases (nad), three for cytochrome c oxidase subunits (coxs), and one for cytochrome b (cob). Transfer RNA genes for 18 amino acids were identified, in which the tRNA genes for leucine and serine are doubled, but the tRNA genes for threonine and valine are not annotated. Phylogenetic analysis using the mitochondrial genomes of seven families of Sphaeropleales indicated that ITBB-AG6 is closely related to *Monoraphidium neglectum*, and falls in the family Selenastraceae with 100% bootstrap support. Two species in the family Neochloridaceae are separated by a species in Hydrodictyaceae, indicating a polyphyletic nature. These findings revealed the complicated phylogenetic relationships of the Sphaeropleales and the necessity of genome sequences in the taxonomy of microalgae.

## Introduction

Chlorophyceae are a genetically, morphologically, and ecologically diverse class of green algae (Leliaert et al. 2012). They are dominant, particularly in freshwater, and plays important roles in global ecosystems (Falkowski et al. 2004). The Chlorophyceae are composed of five taxonomic orders: Sphaeropleales, Chlamydomonadales, Chaetophorales, Chaetopeltidales, and Oedogoniales (Leliaert et al. 2012). Sphaeropleales is one of the major orders in the class Chlorophyceae (Fucikova et al. 2014), and contains some of the most common freshwater species (e.g. Scenedesmus, Desmodesmus, Tetradesmus, and Raphidocelis), including some species used in applications such as bioassays and biofuel production (Krienitz et al. 2003). However, morphologies and 18S rRNAs of the species in Sphaeropleales are similar, resulting in low resolution of conventional classification. Genome-based phylogenetic analysis can produce better resolved trees that reflect the overall relationship between highly related species (Alam et al. 2010; Lemieux et al. 2015). In the class Chlorophyceae, mitochondrial genomes from some genera have been characterized, but mostly limited to the order Volvocales (Denovan-Wright et al. 1998; Kroymann and Zetsche 1998; Smith et al. 2013). Mitochondrial genome data on the sister group of the Volvocales, the order Sphaeropleales, are represented by *Scenedesmus obliquus* (Nedelcu et al. 2000), which was recently recombined as *Acutodesmus obliquu*s (Hepperle et al. 2000). This study sequenced the whole mitochondrial genome of a Sphaeropleales species, *Chlorolobion braunii* (Nägeli) Komárek 1979 strain ITBB-AG6, which was isolated from an azolla community in a sewage pond.

## Materials

**Figure 1.**
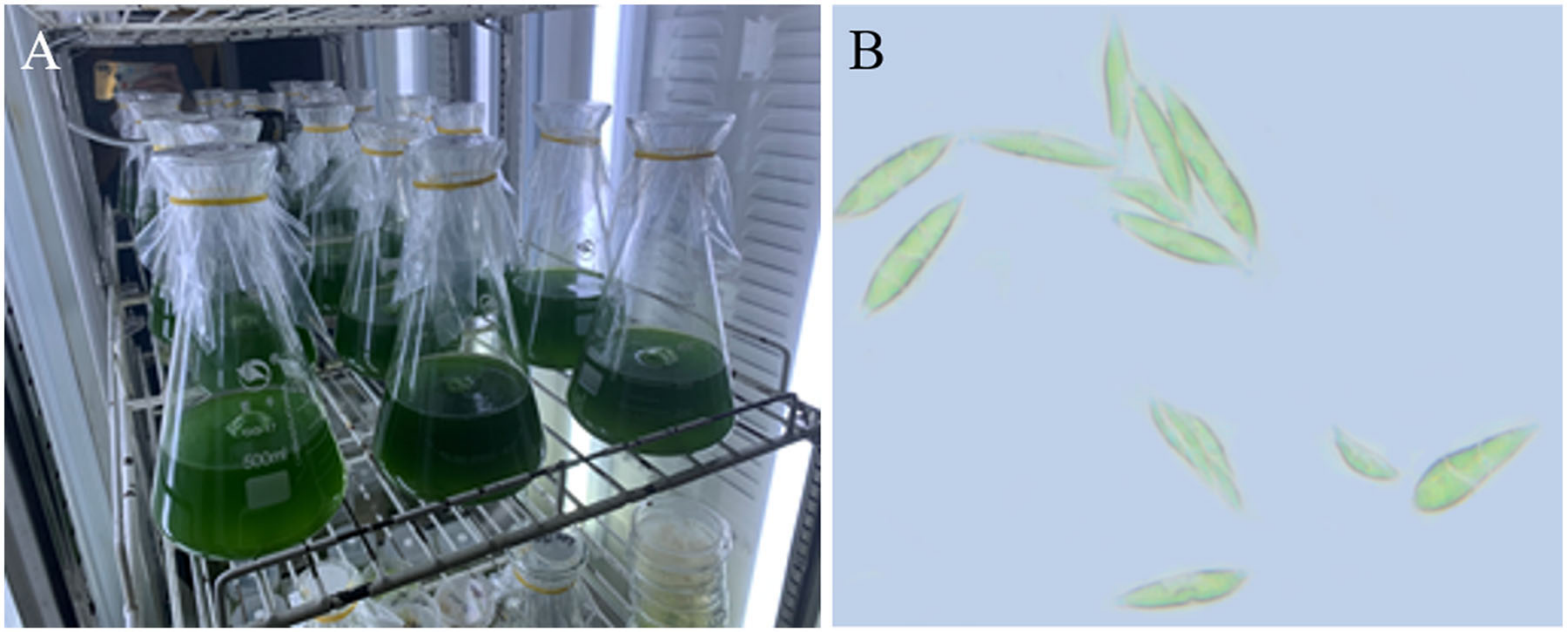
Propagation of the algal strain ITBB-AG6 (A) and its morphology (B). The alga from wild environments is single-celled and straight, long, olive-shaped, or oval under certain culture conditions. The photos were taken by Yiliang He.

*C. braunii* strain ITBB-AG6 was isolated from an azolla community in a sewage pond in Danzhou City, Hainan Province, China (19.5211N, 109.5119E). The species was morphologically identified by referring to the images in the Culture Collection of Algae at Göttingen University, international acronym SAG (https://www.uni-goettingen.de/en/184982.html) and the AlgaeBase (Guiry and Guiry 2018). The strain was cultured in TAP medium (Figure 1) and harvested by centrifugation for DNA isolation. A sample of the culture was stored at the ClonBank of Institute of Tropical Bioscience and Biotechnology at −80 °C in 15% glycerol with voucher number ITBB-AG6 (Curator, Deguan Tan, tandeguan@itbb.org.cn).

## Methods

The genomic DNA was extracted from the biomass of ITBB-AG6 using the Universal Genomic DNA Extraction Kit (Sangon, Shanghai, China) according to the manufacturer’s instruction. The genomic DNA was sequenced using Illumina Hiseq 2500 and Nanopore platforms, and assembled with Canu v1.5 (Koren et al. 2017) and wtdbg2 (Ruan and Li 2020). The scaffolds containing mitochondrial genome were identified by a local blast search using a mitochondrial genome sequence of *Heveochlorella hainangensis* (NC_048968) as a reference (Yu et al. 2020). The sequence was further corrected using pilon (Walker et al. 2014). The overlapped terminal repeat was removed using MacVector 13.6. The quality of the final circular genome was assessed by mapping the Illumina reads to the assembly, and the sequence depth and coverage was estimated using a recently published protocol (Ni et al. 2023). The final genome was annotated with the MITOS webServer (http://mitos2.bioinf.uni-leipzig.de), and manually corrected using MacVector 13.6. Visualization was generated using the Proksee server (https://proksee.ca/), which utilizes GCView (Stothard and Wishart 2005) for circular genome drawing. We also tried to confirm its identity by 18S rDNA sequence, and found that almost all strains of Sphaeropleales available have identical 18S rDNA sequences.

For phylogenetic analysis, mitochondrial genomes of 16 algal species from seven families of Sphaeropleales were retrieved from GenBank. Coding DNA sequences of 12 genes (*atp6*, *atp9*, *cox1*, *cox2*, *cox3*, *nad1*, *nad2*, *nad3*, *nad4*, *nad5*, *nad6*, and *cob*) that were shared by all taxa were extracted and combined in the same order, and aligned by Clustal Omega (Sievers and Higgins 2021). Phylogenetic trees were generated using the maximum-likelihood (ML) methods with 1000 bootstrap replicates in MEGAX (Kumar et al. 2018) and were rooted with a *Chlorella vulgaris* genome (Hu et al. 2020). The evolutionary history was inferred using the ME method (Rzhetsky and Nei 1992). The optimal tree with the sum of branch length = 0.50 is shown in this paper. All positions containing gaps and missing data were eliminated from the dataset (complete deletion option).

## Results

**Figure 2.**
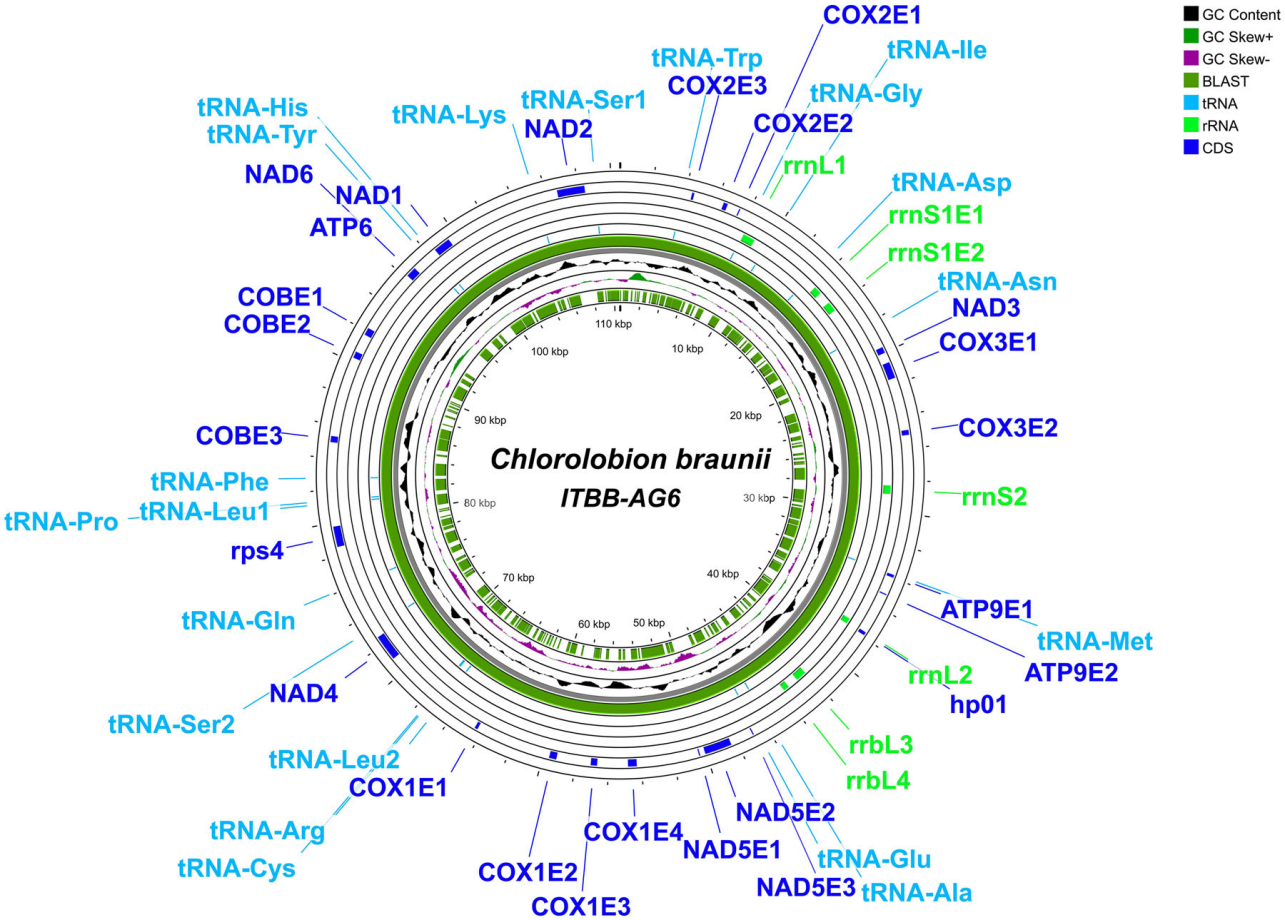
Circular sketch map of the complete mitochondrial genome of *C. braunii* ITBB-AG6. Positions of protein-coding genes, rRNA, and tRNA genes are indicated.

The full mitochondrial genome of *C. braunii* ITBB-AG6 has a length of 110,124 bp and harbors at least 40 genes, including 17 protein-coding genes, 20 tRNA genes, and six rRNA genes (Figure 2). The average coverage depth was 3328× with a minimum depth of 62× and a maximum depth of 29,756× (Supplementary Figure S1). The overall GC content is 48.4% with a ratio of A:G:C:T = 26.0:24.5:23.9:25.6. The protein-coding genes include two for ATP synthases, seven for NAD(P)H-quinone oxidoreductases (nad), three for cytochrome c oxidase subunits (cox), and two for cytochrome b (cob) and rps4. Both the ribosomal RNA genes for the large and small subunits were fragmented. The large subunit RNA gene was divided into four fragments (rrnL1, rrnL2, rrnL3, and rrnL4), whereas the small subunit RNA gene was divided into two fragments (rrnS1 and rrnS2). Transfer RNA genes for 18 amino acids were identified, in which the tRNA genes for leucine and serine were doubled, whereas the tRNA genes for threonine and valine are missing.

**Figure 3.**
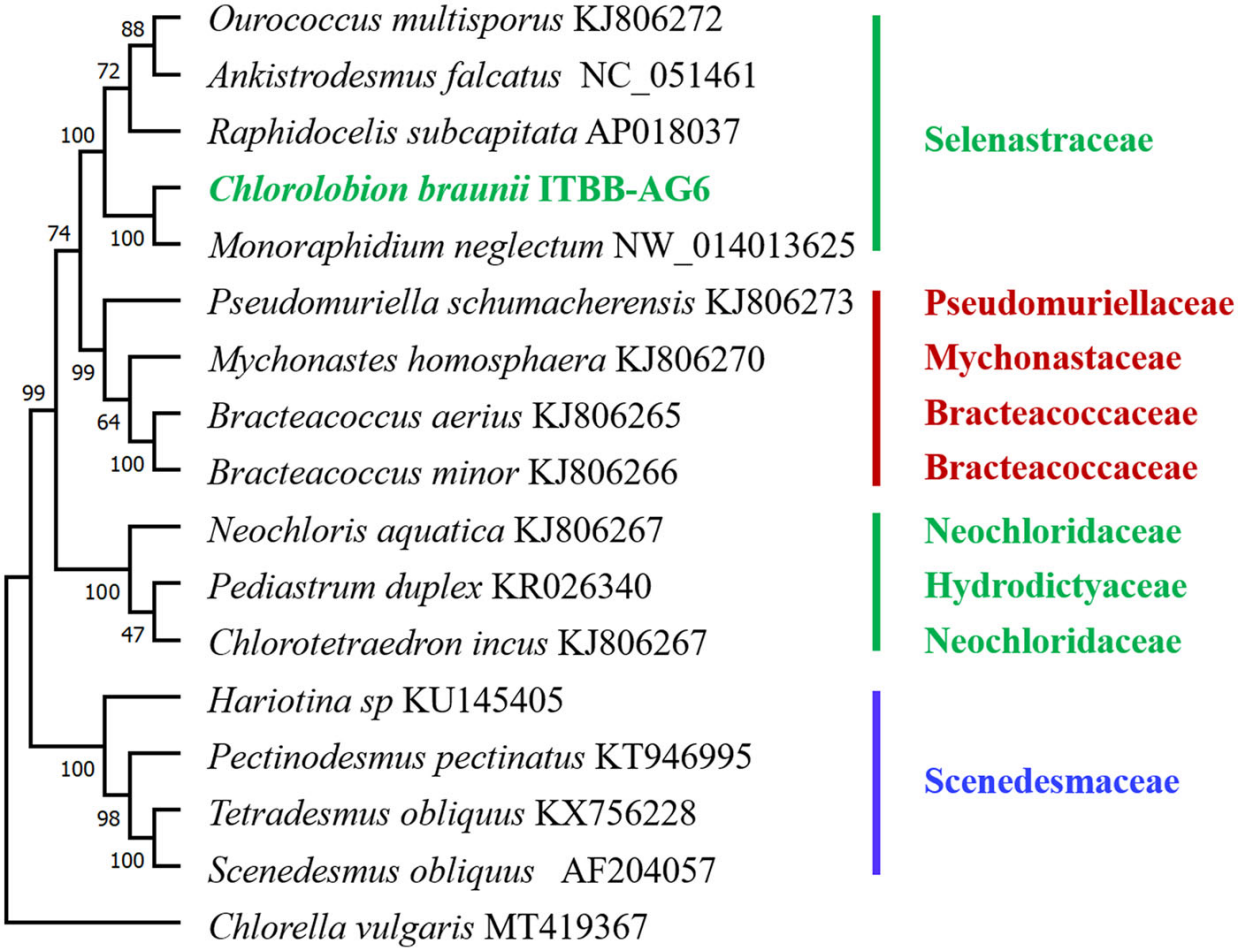
Maximum-likelihood tree of algal species in Sphaeropleales (Chlorophyta). The tree was rooted with a *Chlorella vulgaris* genome (Hu et al. 2020). Bootstrap supports (1000 replicates) for clades are shown above or below the branches.

Phylogenomic analysis using the coding DNA sequences of 12 genes shared by related species in Sphaeropleales revealed that strain ITBB-AG6 is most closely related to *Monoraphidium neglectum* (Bogen et al. 2013), and falls in the family Selenastraceae with 100% bootstrap support (Figure 3). *C. braunii* was once named as *Monoraphidium braunii* (Komárková-Legnerová 1969) and *Rhaphidium braunii* (Komárek 1979), suggesting the difficulty in conventional classification. The families Selenastraceae and Scenedesmaceae are monophyletic in the phylogeny (Figure 3). However, two species in the family Neochloridaceae are separated by a species in Hydrodictyaceae, indicating the polyphyletic nature of this family and the necessity of genome-based phylogenetic analysis in the taxonomy of microalgae.

## Discussion and conclusions

Owing to the significant application prospects in biodiesel production and sewage treatment, the Sphaeropleales have attracted extensive research. Chloroplast genomes of many species have been extensively analyzed (de Cambiaire et al. 2006; Lemieux et al. 2015; Fucikova et al. 2016a, 2016b; He et al. 2018). However, the mitochondrial genomes remain relatively uncharacterized. This research reports the mitochondrial genome of *C. braunii* ITBB-AG6, which supports the evolutionary relationship of subfamilies in Sphaeropleales. It also provides a basis for future molecular biology studies and fills in the data gaps on population structure and genetic diversity within its geographic range. Simultaneously, we found by analysis of mitochondrial annotation data that the tRNA genes for leucine and serine were doubled, but the tRNA genes for threonine and valine were not annotated. Therefore, tRNA-Thr and tRNA-Val may be encoded by nuclear genes and transported into mitochondrion as many species did (Sharma and Sharma 2015; Kulkarni et al. 2021). Most algal species in Selenastraceae such as *M. neglectum*, *O. multisporus*, and *A. falcatus* did not seem to have the tRNA-Thr gene in their mitochondrial genomes, either, but they had the tRNA-Val genes.

Traditional classification of unicellular green algae was often problematic due to the lack of significant morphological characteristics. Phylogenetic analysis using genetic barcodes such as the18S rRNA gene sequences were often used to resolve the problem (Ma et al. 2013, 2015; Sanders et al. 2016). However, the 18S rRNA sequences are highly conserved and almost identical in Sphaeropleales, resulting in low resolution of conventional classification. Genome-based phylogenetic analysis may be a better solution (Alam et al. 2010; Lemieux et al. 2015). In this research, based on the coding DNA sequences of 12 genes shared by related species in Sphaeropleales, we revealed that strain ITBB-AG6 is most closely related to *M. neglectum* (Figure 3), and that the families Selenastraceae and Scenedesmaceae are monophyletic, whereas Neochloridaceae are polyphyletic.

In summary, the mitochondrial genome of *C. braunii* ITBB-AG6 was sequenced, assembled, and annotated. This study provides detailed basic molecular data for species identification, bioenergy, and sewage treatment industry.

## Supplementary Material

Supplemental Material

## Data Availability

The genome sequence data that support the findings of this study are openly available in GenBank of the NCBI at https://www.ncbi.nlm.nih.gov under the accession no. OP778184. Raw reads generated by the authors to assemble this mitochondrial genome are available under GenBank BioProject no. PRJNA931121, BioSample no. SAMN33050851, and SRA no. SRR23329251.
