# Supplementary figures and images for: Characterization of the mitochondrial genome of Chlorolobion braunii ITBB-AG6, an azolla-associated green alga isolated from sanitary sewage
Source: Mitochondrial DNA B Resour. 2023 Aug 3;8(8):826–30. doi: 10.1080/23802359.2023.2241573 (PMC10402853; doi:10.1080/23802359.2023.2241573)

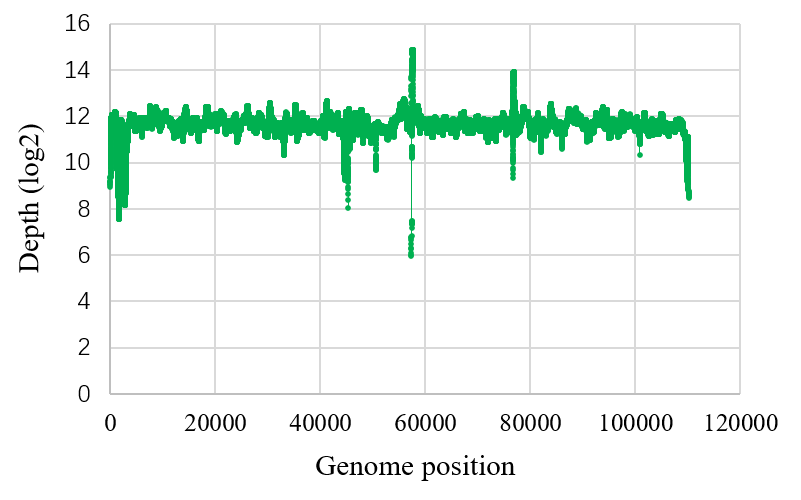

Supplement: Supplemental Material [file TMDN_A_2241573_SM5594.tif]
